# Optimising realised genetic gain with low forward predictive ability across cycles for cooking time and correlated traits in common bean based on multivariate genomic selection

**DOI:** 10.1007/s00122-026-05263-0

**Published:** 2026-06-19

**Authors:** Renu Saradadevi, Winnyfred Amongi, Clare Mukankusi, Allan S. Male, Jean-Claude Rubyogo, Eric Huttner, Felipe A. Castro-Urrea, Li Li, Kadambot H. M. Siddique, Wallace A. Cowling

**Affiliations:** 1 The UWA Institute of Agriculture, The University of Western Australia, Perth, WA 6009 Australia; 2 UWA School of Agriculture and Environment, The University of Western Australia, Perth, WA 6009 Australia; 3 Alliance of Bioversity International and International Center for Tropical Agriculture (CIAT), c/o National Agricultural Research Laboratories, Kawanda, P.O. Box 6247, Kampala, Uganda; 4 Alliance of Bioversity International and International Center for Tropical Agriculture (CIAT), ICIPE Campus, Duduville Complex, Kasarani Road, P.O Box 823, Nairobi, 00621 Kenya; 5 Present Address: Chloroplast Consulting, Canberra, ACT 2614 Australia; 6 Australian Centre for International Agricultural Research, Forrest, ACT 2617 Australia; 7 Animal Genetics and Breeding Unit, The University of New England, Armidale, NSW 2351 Australia; 8 Present Address: NPZ Australia Pty Ltd, 1 Underwood Avenue, 6008 Shenton Park, Australia

## Abstract

**Key message:**

Rapid genetic gain was achieved for cooking time in common bean based on multivariate genomic analysis, but forward predictive ability was low and phenotyping remains essential to secure genetic gain.

**Abstract:**

Common beans (*Phaseolus vulgaris* L.) are a major source of protein and energy in sub-Saharan Africa, but their long cooking time (CKT) imposes social, economic, environmental and health burdens. This study aimed to accelerate genetic gain for shorter CKT while maintaining or improving other seed traits such as seed iron (Fe) and zinc (Zn) content, water absorption capacity (WAC) and 100-seed weight (SW100) across rapid cycles of early-generation genomic selection. Two related founder populations were selected from the African bean panel and intercrossed in 2020 (population A) and 2021 (population B), followed by rapid two-year cycles of augmented S_0_-derived family selection based on an index and optimal contributions selection. Best linear unbiased predictions (BLUPs) of breeding values were obtained from pedigree (ABLUP), genomic (GBLUP) and single-step (HBLUP) multivariate linear mixed model analysis across two cycles. Realised genetic gain from cycle 1 to cycle 2 was high for CKT (average -8.0 min *y*^−1^) and favourably correlated with WAC (average + 7.7% *y*^−1^), but detrimental genetic correlations limited gain in Fe, Zn and SW100. Genomic and single-step models resulted in high accuracy of BLUPs based on prediction error variance. Forward predictive ability for CKT across cycles was low, but rank correlation of realised and predicted HBLUPs for CKT in cycle 2B S_0_ seedlings was moderate-high (0.734) when phenotypes for Fe, Zn, WAC and SW100 in cycle 2B were included in the analysis. Desired genetic gains in CKT, Fe and Zn in future cycles will require high levels of phenotyping in each cycle, although easy-to-measure correlated traits such as WAC and SW100 may assist genetic gain in all traits.

**Supplementary Information:**

The online version contains supplementary material available at 10.1007/s00122-026-05263-0.

## Introduction

Common bean (*Phaseolus vulgaris* L.) is a staple food in Africa and plays a crucial role in providing essential nutrients and sustaining traditional diets due to its high levels of grain protein and carbohydrate, and moderate levels of iron (Fe) and zinc (Zn). However, traditional bean varieties pose significant challenges because of their long cooking time (CKT), which is a major disincentive to consumption and processing. This challenge is particularly concerning in sub-Saharan Africa, where more than 80% of households rely on polluting fuels such as wood, charcoal, coal or kerosene for cooking (Stoner et al. 2021). The long cooking time for beans exposes women and children to prolonged smoke inhalation with negative health outcomes (Fullerton et al. 2008). Additionally, gathering fuel for cooking contributes to deforestation, greenhouse gas emissions and environmental degradation (Bailis et al. 2015; Stoner et al. 2021), and the collection of firewood by women and children exposes them to personal risks and is a time-consuming activity (Shellie-Dessert & Hosfield, 1990). The high fuel and water demand for cooking beans discourages consumption despite their nutritional value (Jeffery et al. 2025). Faster cooking bean varieties with improved canning quality traits may therefore promote bean consumption and processing in Africa (Kesiime et al. 2024).

To address these challenges, we initiated a rapid cycle genomic selection (GS)-based breeding programme for common bean in Uganda in 2019 (Saradadevi et al. 2021). The aim was to reduce CKT and improve seed Fe and Zn content, along with higher grain yield (GY) and market appeal in major bean market groups in East Africa. Founder parents were crossed in two rounds to establish two populations (A and B) in 2020 and 2021. We used a rapid cycle early-generation selection method developed in maize known as S_0_-derived family selection (Walsh and Lynch 2018) in combination with a form of optimal contribution selection (OCS) (Woolliams et al. 2015) where the mating list is optimised to achieve the desired balance between next-generation genetic gains and parental coancestry (Kinghorn 2011). OCS is based on an index derived from the sum of breeding values for each trait weighted to achieve desired genetic gains (Brascamp 1984; Saradadevi et al. 2021). We introduced the acronym BRIO to describe this breeding framework in self-pollinating crops which emphasises the importance of accurate breeding values (B), rapid breeding cycles (R), index selection (I) and optimal mating designs based on OCS (O) (Cowling et al. 2023; Saradadevi et al. 2021).

In S_0_-derived family selection, noninbred individuals are used as parents to begin the next cycle, which potentially shortens breeding cycles compared to traditional selection on inbred lines. Selection on noninbreds may increase the rate of genetic gain by hastening selection cycles (Cobb et al. 2019). Breeding values of S_0_ individuals for complex traits normally are not based on their own phenotypes and instead are based on phenotypes measured in their S_0,1_ or S_0,2_ self-bulks (Walsh and Lynch 2018). In common beans and most annual crop plants, crossing cannot be achieved normally on S_0_ plants because breeding values are assigned after maturity; in that case, crossing must occur on plants grown from remnant S_0,1_ or S_0,2_ seeds. In augmented S_0,1_ family selection, S_1_ parent plants contribute both cross- and self-progeny to the next breeding cycle, which enhances genetic relationships across cycles, and if such progeny are selected for crossing in the next cycle, this may generate near-inbred individuals for commercial evaluation (Cowling et al. 2023). This approach has proven effective in self-pollinating crops such as oilseed rape and field pea where accurate breeding values in noninbred progeny were achieved for moderately heritable traits, and S_0_ individuals with superior breeding values were selected (as S_0,1_ or S_0,2_ plants) for crossing to begin the next cycle, thereby reducing cycle length and potentially accelerating genetic gain (Castro-Urrea et al. 2023; Cowling et al. 2023).

Linear mixed model (LMM) equations generate best linear unbiased predictions (BLUPs) of breeding values of individuals with pedigree relationship information (ABLUPs) or genomic relationship information (GBLUPs). ABLUPs obtained in augmented S_0,1_ family selection in oilseed rape and field peas were accurate for S_0_ individuals (*r* > 0.8) and were only a few percentage points lower than accuracy in S_2_ or higher inbred progeny for several low-medium heritability traits (Castro-Urrea et al. 2023; Cowling et al. 2023). The achievement of accurate breeding values in noninbred progeny permits earlier selection and lower breeding costs within cycles as demonstrated in oats (Mellers et al. 2020), and potentially shorter breeding cycles as demonstrated in field peas (Castro-Urrea et al. 2023) and oilseed rape (Cowling et al. 2023).

Multivariate genomic analysis enables simultaneous evaluation of multiple traits while explicitly estimating genetic correlations among them (Endelman 2023; Klápště et al. 2020). Genetic correlations are central to understanding the degree to which traits share genetic architecture, which in turn allows breeders to exploit favourable associations, anticipate trade-offs due to unfavourable associations, and design more efficient selection strategies (Falconer and Mackay 1996). By integrating information across traits and exploiting the additive genetic correlations among them, multivariate LMM can potentially increase the accuracy of estimated breeding values, especially for traits that are low-to-moderately heritable or sparsely measured (Pszczola et al. 2013). The benefits of multivariate LMM are particularly evident when phenotypic data are missing for one trait but are available for correlated traits, because the shared information enhances the prediction accuracy of the non-phenotyped individuals (Calus and Veerkamp 2011; Pszczola et al. 2013). This was the case in multivariate *vs* univariate LMMs in the analysis of low-to-medium traits in non-inbred individuals of field pea (Castro-Urrea et al. 2023).

One of the traits that may benefit from multivariate LMM in common beans is CKT, which is associated with other seed traits such as water absorption capacity (WAC) and 100-seed weight (SW100) (Cichy et al. 2015; Diaz et al. 2021; Elia et al. 1997). CKT is measured in Mattson cookers (Proctor and Watts 1987) and is time-, cost- and labour-intensive. With more than a thousand samples per breeding cycle and very limited seed availability in early generations of our common bean breeding programme, replication of samples was not feasible, and we expected low reliability of phenotyping for CKT. We used multivariate LMM to exploit potential correlations of CKT with easier-to-measure traits such as WAC and SW100. Potentially, multivariate LMM based on correlated traits will enhance prediction accuracy of traits which have low heritability, as achieved in perennial tree breeding (Klápště et al. 2020).

In annual crop breeding programmes, individuals may be phenotyped and their pedigree recorded, but they may lack genotypic data due to budget constraints, technical failures in DNA extraction, sample contamination, or filtering to remove individuals which do not meet the minimum requirements for successful SNP allele identification. This was the case in our common bean breeding programme in East Africa, and therefore, it was important to explore the value of single-step genomic evaluation which combines the pedigree and genomic matrices into an H-matrix to estimate HBLUPs (Aguilar et al. 2010), also known as ssGBLUPs. Also, we relied on a modest and affordable SNP panel (Ariza-Suarez et al. 2023) that may lack SNPs linked to some beneficial alleles, and we expected that this would reduce reliability of genomic predictions (Meuwissen et al. 2001). In this situation, analysis with the H-matrix may improve accuracy of breeding values and predictive ability across cycles (Aguilar et al. 2010; Legarra et al. 2009).

Single-step genomic evaluation with HBLUPs achieved higher predictive ability compared to ABLUPs or GBLUPs in Danish Duroc pigs (Christensen et al. 2012). Single-step multivariate genomic analysis improved the accuracy of breeding values over univariate analysis in USA Holstein cattle (Aguilar et al. 2011). In plant breeding programmes, single-step models outperformed genomic models under partial phenotyping scenarios where a subset of individuals within the population were phenotyped (Ashraf et al. 2016; Pérez-Rodríguez et al. 2017; Sood et al. 2020). Isik et al. (2026) recently implemented ssGBLUP in genomic prediction across generations in loblolly pine, but the challenge remains to implement across-generation validation in self-pollinating crops. However, forward prediction across cycles remains challenging in self-pollinating crops which have low predictive accuracy as a result of disrupted genetic relationships between families and different environments in each cycle (Sun et al. 2019).

The primary objective of this study was to evaluate multivariate single-step genomic analysis to measure realised genetic gain across rapid cycles of S_0_-derived family selection for CKT, Fe, Zn, WAC and SW100 in common bean, and to assess forward predictive ability for these traits across breeding cycles. Multivariate LMMs can reveal genetic correlations among traits in a timely manner, so that strategies to combat detrimental genetic correlations may be formulated and implemented. While the statistical approaches used in this study are well established, their performance under operational breeding of self-pollinating crops, particularly across multiple cycles and multiple traits that are costly and difficult to phenotype, remains less well documented. In practical breeding programmes, decisions are often made using incomplete, unbalanced, and early-generation data, where traits such as CKT, Fe, Zn and WAC are measured on limited seed quantities and without replication. Under such constraints, the effectiveness of multivariate genomic models, and their ability to support selection through correlated traits, is an empirical question rarely addressed in crop breeding. Therefore, this study explores the option of multivariate genomic analysis combined with OCS on an index of multiple traits to optimise genetic gain through rapid cycles of early-generation selection in a self-pollinating crop.

## Materials and methods

### East African bean breeding programme

This common bean breeding programme began in 2019 at the Alliance of Bioversity International and CIAT (Alliance) in Kampala, Uganda, in partnership with national agricultural research and extension systems (NARES) in six East African countries who are collaborators in the Pan-African Bean Research Alliance (PABRA): Uganda (UGA), Tanzania (TZA), Kenya (KEN), Rwanda (RWA), Burundi (BDI) and Ethiopia (ETH). The aim of the programme was to accelerate genetic gain in grain yield (GY) while decreasing CKT, increasing WAC and seed Fe and Zn, and maintaining SW100 within common bean market groups in East Africa (Saradadevi et al. 2021).

**Fig. 1:**
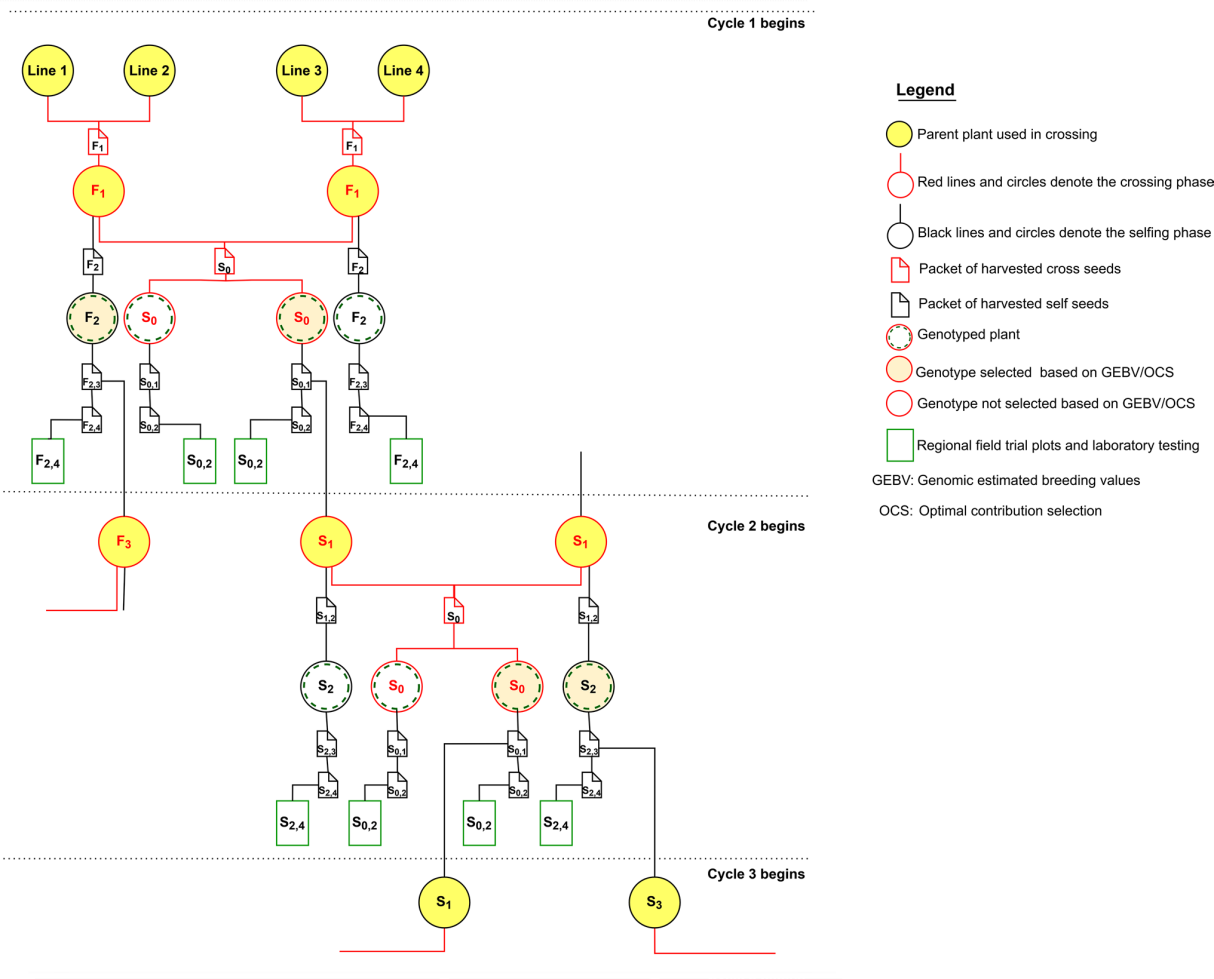
Diagram of augmented S_0,1_ and S_0,2_ family selection in the breeding programme of common bean over two cycles. Individuals in cycle 2 included S_0_ cross-progeny augmented with S_2_ selfs of S_1_ parent plants. In cycle 2, S_0_ and S_2_ progeny plants were genotyped and phenotypes were derived from their self-families which provided an estimate of the breeding value of those individuals (Walsh and Lynch 2018)

We follow nomenclature for progeny generations used in maize breeding when the segregating progenies are derived from heterozygous parent plants and are named S_0_ generation, and from self-pollinating crop breeding when the non-segregating progenies are derived from homozygous parent plants and are named F_1_. A rapid cycle early-generation recurrent selection programme was implemented, based on S_0,1_ family selection in maize, where S_0_ denotes a cross-progeny from heterozygous parent plants and S_0,1_ a bulk of self-seed from an S_0_ plant. In S_0,1_ family selection, several S_0,1_ plants are phenotyped and their mean value provides an estimate of the breeding value of the S_0_ mother plant (Walsh and Lynch 2018). We used augmented S_0,1_ family selection, where S_1_ parent plants contributed both self- and cross-progeny to the next cycle (Fig. 1) (Cowling et al. 2023).

Crossing was carried out at the beginning of each calendar year of the breeding programme, based on an optimised crossing list which was developed as follows: (i) Data accumulated across cycles of selection were analysed by multivariate LMM in software ASReml-R v4.2 (Butler et al. 2023) to derive BLUPs for traits; (ii) BLUPs were weighted in a selection index for desired genetic gains in software DESIRE (Kinghorn 2025), where index weights were derived to achieve predefined genetic gains across traits as described in Saradadevi et al. (2021); and (iii) the indices for all genotypes in the pedigree were submitted to OCS in software MateSel (Kinghorn 2011), with weightings and constraints easily invoked to ensure practical relevance, precise control of the response to each trait in the economic index and other requirements of progressive breeding programmes (Kinghorn et al. 2011; Kinghorn and Kinghorn 2025), following procedures outlined previously (Cowling et al. 2023; Saradadevi et al. 2021).

#### *Population A*

Founders for crossing to begin cycle 1 in population A (cycle 1A) were selections for GY, CKT, Fe and Zn in a restricted African bean panel composed of 161 accessions with SNP genotypic data and phenotypic data for GY, CKT, Fe, Zn available from four field trials in East Africa in 2014 and 2015 (unpublished data, Alliance). In a two-stage analysis, BLUEs were derived from field and laboratory data for each trait; then, BLUEs were submitted with SNP genotypic data to a multivariate LMM to predict GBLUPs for each candidate accession. GBLUPs were added to an economic index, weighted for desired genetic gains, and the index was submitted to OCS in software MateSel. A crossing list was chosen with 91 founder parents in 120 crosses to begin cycle 1A in 2020.

#### *Population B*

The African bean panel was expanded to 356 accessions with additional SNP genotypic data and phenotypic data for GY, CKT, Fe and Zn from 33 field trials in East Africa (Saradadevi et al. 2021). A second group of founder parents were selected from the panel for crossing in 2021 to begin cycle 1 in population B (cycle 1B), based on a two-stage analysis of genotypic and phenotypic data in the 356 accessions (Saradadevi et al. 2021). GBLUPs were added to an economic index, weighted for desired genetic gains, and the index was submitted to OCS in software MateSel as outlined above. A crossing list was chosen with 88 founder parents in 120 crosses to begin cycle 1B in 2021.

#### *Cycle 1 of S*_*0,1*_* and S*_*0,2*_* family selection*

**Fig. 2:**
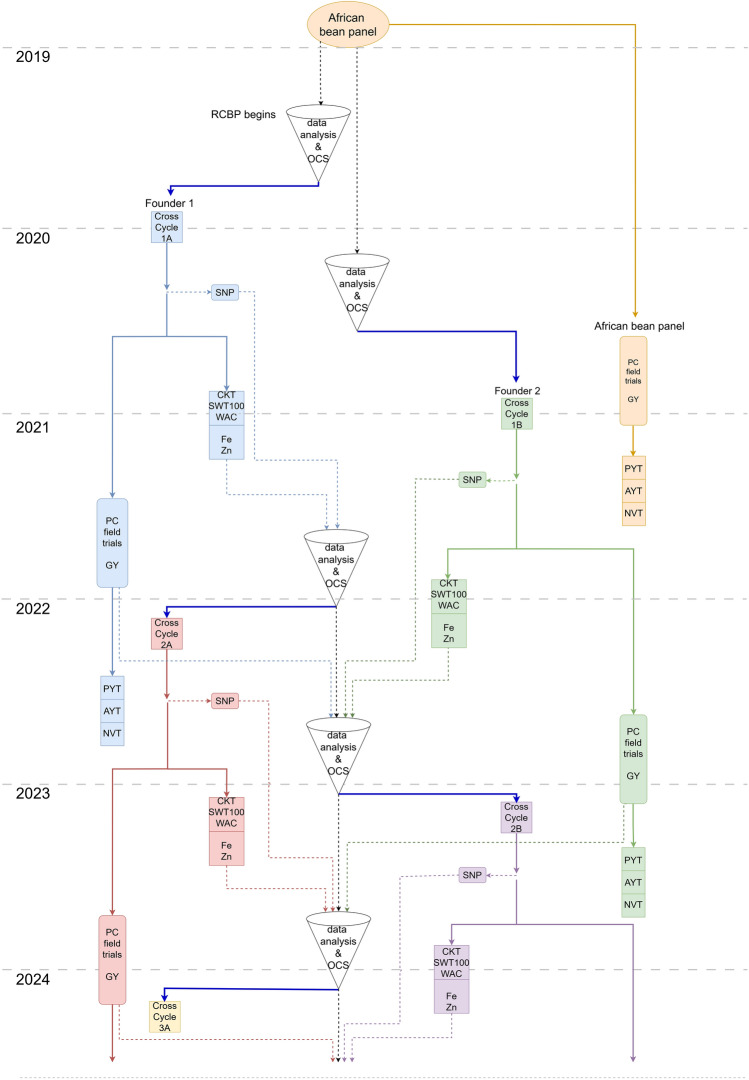
Flow chart of the common bean breeding programme from 2019 to 2024, showing flow of data (broken arrows) and seed (solid arrows). Cycles 1A, 1B, 2A and 2B are shown in distinct colours. Solid blue arrows represent the generation of an optimised crossing list with optimal contributions selection (OCS) based on analysis of all available data at that time. Abbreviations: single-nucleotide polymorphism (SNP), rapid cooking bean project (RCBP), partner country (PC), preliminary yield trial (PYT), advanced yield trial (AYT), national variety trial (NVT), grain yield (GY), cooking time (CKT), 100-seed weight (SW100), water absorption capacity (WAC), seed iron (Fe), seed zinc (Zn)

To initiate cycle 1A (population A) in 2020, the F_1_ progeny of crosses among near-homozygous bean varieties in population A were intercrossed in a pairwise fashion to generate segregating heterozygous (S_0_) progeny plants. To initiate cycle 1B (population B) in 2021, the F_1_ progeny of crosses derived from OCS within population B were intercrossed in a pairwise fashion to generate S_0_ progeny plants. Each F_1_ parent plant was also selfed to generate F_2_ seeds, which were taken forward for genotyping and phenotyping along with S_0_ cross-progeny (Fig. 1). Individual S_0_ and F_2_ progeny plants were grown and genotyped by leaf sampling as described below, and S_0,1_ and F_2,3_ self-family bulks were generated from each individual (Fig. 1). Five plants were selfed and bulked from each S_0,1_ to generate S_0,2_ bulks, and five plants from each F_2,3_ to generate F_2__,__4_ bulks. Self-family bulks were phenotyped for CKT, Fe, Zn, SW100 and WAC in the seed laboratory (see methods below) and for GY in field trials in six countries in East Africa (Fig. 2). Laboratory data were obtained on S_0,1_ family bulks within 12 months of crossing, and GY and field data on S_0,2_ family bulks within two years of crossing (Fig. 1, Fig. 2). This study focuses on the analysis of the laboratory data.

#### *Cycle 2 of S*_*0,1*_* and S*_*0,2*_* family selection*

A similar process was undertaken to begin cycle 2 within populations A and B, but in this case most of the parents were S_0,1_ plants grown from retained S_0,1_ self-seed of S_0_ progeny of cycle 1 (Fig. 1). The breeding design was based on S_0,1_ family selection (Walsh and Lynch 2018) which was modified to include S_1,2_ self-progeny of the S_1_ parent plants as candidates in the next cycle. This was previously defined as augmented S_0,1_ family selection (Cowling et al. 2023) (Fig. 1).

In population A, genotypic data and phenotypic data on CKT, Fe, Zn, SW100 and WAC from cycle 1A progeny were available to analyse and initiate cycle 2A crossing in early 2022 (Fig. 2). A two-stage analysis was conducted on laboratory traits and GY as described in Saradadevi et al. (2021) to generate GBLUPs in a multivariate LMM with a genomic relationship matrix. An optimised crossing list was generated in MateSel based on an index of multiple traits. In early 2022, retained seeds of self-families of selected genotypes were sown as parent plants to initiate cycle 2A with a target of 120 crosses based on OCS implemented in MateSel (Fig. 2). The cycle 2A S_0_ progeny and S_2_ selfs of S_1_ parent plants were genotyped and their self-families phenotyped for CKT, Fe, Zn, SW100 and WAC in the laboratory in 2022 and for GY in the field in 2023 (Fig. 1, Fig. 2).

In population B, crossing to initiate cycle 2B was carried out in 2023 with parent selection following the same approach as for cycle 2A. Two distinct end-use preferences were incorporated into the OCS software MateSel. The first end-use prioritised fast cooking time with moderate Fe, and the second prioritised high Fe with moderate CKT. This strategy was intended to address unfavourable genetic correlations observed between CKT and Fe/Zn content during analysis of cycle 1B data.

The data used in analysis in this study include genotypic and phenotypic records from all individuals in cycles 1A, 1B, 2A and 2B from the laboratory, including CKT, Fe, Zn, SW100 and WAC.

### Pedigree records

The pedigree of each individual was entered into a database, Breeding Management System (BMS) (https://bmspro.io/), by recording the name of the individual, its male and female parent names, and the number of selfing generations (fgen) to generate that individual. For an S_1_ individual, fgen was recorded as 0 because the male and female parents of the S_1_ were explicitly recorded as the same S_0_ individual. For an inbred ancestral or founder variety, fgen was recorded as 6 on the assumption that the male and female parents were near-homozygous. The pedigree of the founder parents was up to five generations deep. The order of individuals in the pedigree file was sorted so that the parents always preceded the progeny.

### SNP genotypic records

In each cycle, leaf samples were collected from each S_0_ cross-progeny plant and each S_1,2_ self-progeny plant (Fig. 1), freeze-dried and sent for genotyping to Diversity Arrays Technology (DArT), Australia. Genotyping was performed using a common bean mid-density panel set of 1861 SNP containing target sites that were used for amplicon sequencing within the DArTag pipeline (Ariza-Suarez et al. 2023). The marker panel was developed from whole-genome re-sequencing and genotype by sequencing of over 1700 breeding lines and landraces from Africa and the Americas (Ariza-Suarez et al. 2023). The refined mid-density SNP panel was derived from an initial dataset of over 40 million SNPs after filtering and removing replicates and defective markers. This panel was designed to be highly informative for various traits, including drought and heat tolerance, pest and disease resistance, cooking time, and genetic diversity (Ariza-Suarez et al. 2023).

### Genomic information to detect related founder parents and inadvertent selfs

Whole-genome SNP data in the common bean mid-density panel were used to discover founder parents that were closely related, and inadvertent selfs of parent plants which arose during emasculation and cross-pollination. Closely related founder parents were identified by the relationship of their SNP genotypes to other founder parents. Inadvertent selfs were identified by comparing the SNP genotype of cross-progeny with the genotype of the mother founder parent and the absence of unique SNPs from the father founder parent.

In cycle 1, whole-genome SNP data of putative S_0_ progeny of F_1_ x F_1_ crosses were compared to SNP data of founder parents and selfs of F_1_ plants, if available. In cycle 2, the SNP genotypes of S_0_ progeny plants were compared to the SNP genotypes of their cross-and-self-sibs, that is, S_2_ self-progeny of the same S_1_ parent plant. Based on their SNP profiles, some putative cross-progeny were deemed to be very similar genetically to their S_1_ mother plant and it was clear that cross-pollination had not occurred as expected; these individuals were classified as inadvertent selfs of the mother plant. Pedigree records were adjusted to accurately reflect these findings.

In the absence of SNP data for either of the parents, or if the parents were strongly related, then it was not possible to deduce whether the alleles were inherited from the male or female parent. In such scenarios, pedigree information was not altered.

### Phenotypic records

In this study, the term *individual* refers to an individual in the pedigree which may or may not be genotyped; this individual may be noninbred, selfed for various generations, or it may be a near-homozygous variety as is normally the case in founder parents, local checks or benchmark varieties (PABRA-recommended control varieties representing different market classes prevalent in East Africa) (Online Resource 1). Phenotypes were measured on the self-family bulks of individuals (Fig. 1) in cycles 1A, 1B, 2A and 2B, and several founder lines and benchmark varieties were included in laboratory phenotyping.

#### *CKT, WAC, SW100, Fe and Zn in the laboratory*

**Fig. 3:**
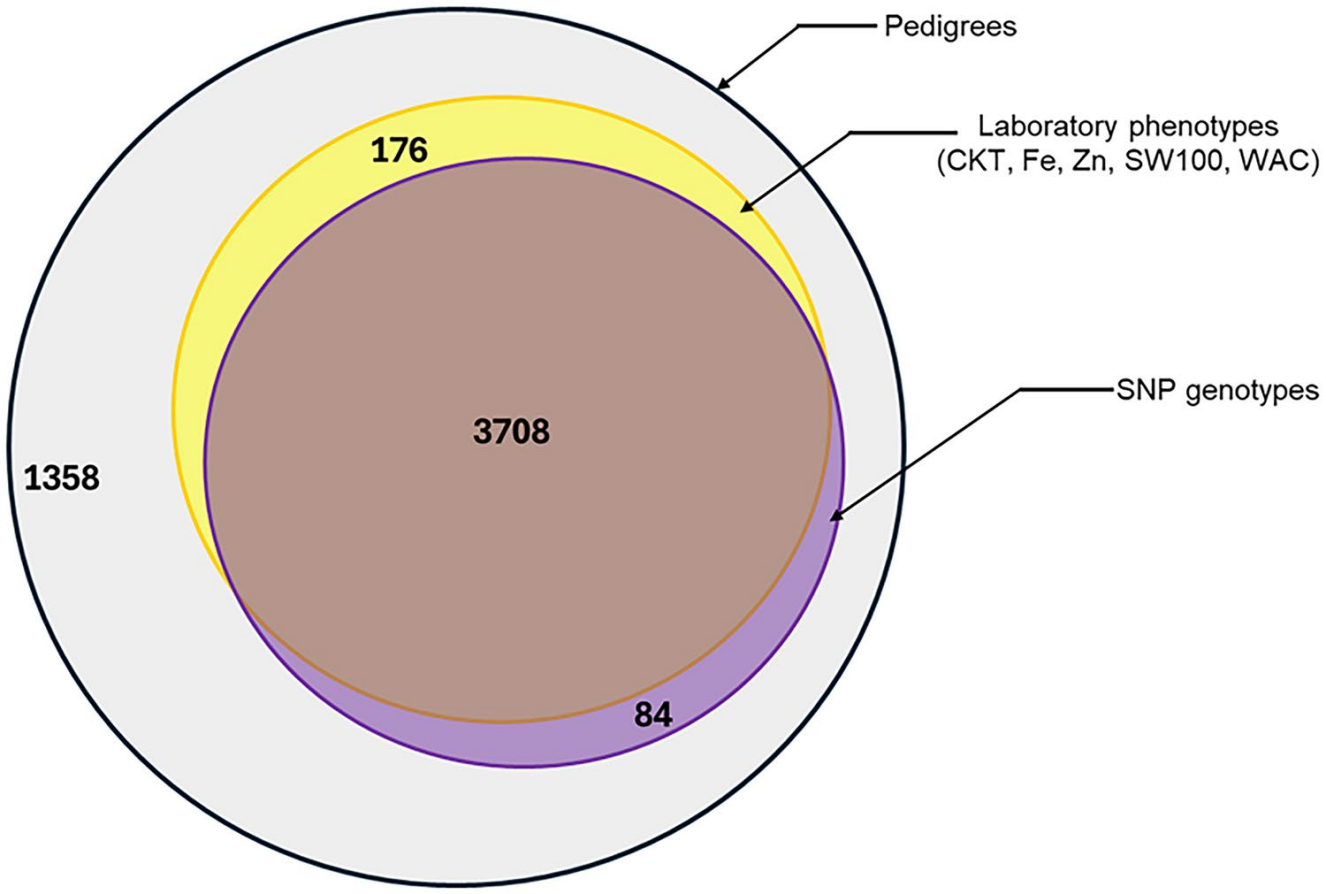
Venn diagram showing number of common bean individuals in the breeding programme combined across populations 1A, 1B, 2A and 2B with pedigrees, SNP genotypes, and laboratory-based phenotypes for cooking time (CKT), iron (Fe), zinc (Zn), 100-seed weight (SW100) and water absorption capacity (WAC)

The S_0_- and F_2_-derived self-family bulks of 3884 individuals, derived from single plants grown in screenhouses at Kawanda, Uganda, were evaluated in the laboratory for CKT, Fe, Zn, SW100 and WAC in cycles 1A, 1B, 2A and 2B (Fig. 3), noting that seed production conditions differed across cycles in terms of date of harvest, timing of seed storage and environmental factors during growth in the screenhouses. Both S_0_- and F_2_-derived materials contributed to the analytical dataset, selection pipeline and early-cycle evaluation. All generations were jointly analysed using multivariate models, where pedigree and genomic relationships across generations were used to estimate breeding values and assess predictive ability. Within years, individuals were chosen for testing in random order.

All laboratory experiments were conducted at the Alliance, Kawanda, UGA. Twenty-five seeds were randomly picked from each self-family bulk to measure CKT using automated Mattson cookers (Canadian Grain Commission, Winnipeg, Canada) (Proctor and Watts 1987). The air-dry weight of 25 seeds of each genotype before soaking was recorded (DryWt). DryWt of 25 seeds was converted to SW100 by multiplying by four. Seeds were soaked in distilled water for 18h in a refrigerator at 5 ºC to provide a consistent time and temperature for soaking, and to avoid the potential impact of fluctuating temperature in the laboratory, as high temperatures during soaking may delay cooking in some varieties (Koriyama et al. 2017). However, this may influence the absolute values of WAC and CKT compared to standard room-temperature protocols used in other studies. Therefore, caution is warranted when comparing absolute values from this study with those reported under different soaking conditions, although relative differences among genotypes are expected to remain consistent at all soaking temperatures. After soaking, the 25 seeds were pat-dried and weighed to record the weight of soaked seeds (SoakWt). Water absorption capacity (WAC) was calculated as the percentage uptake of water by dry seeds as shown in Eq. 1.

1 $$\mathrm{W}\mathrm{A}\mathrm{C} \left(\%\right)=\frac{\mathrm{S}\mathrm{o}\mathrm{a}\mathrm{k}\mathrm{W}\mathrm{t}-\mathrm{D}\mathrm{r}\mathrm{y}\mathrm{W}\mathrm{t}}{\mathrm{D}\mathrm{r}\mathrm{y}\mathrm{W}\mathrm{t}}*100$$

The soaked seeds were then placed in the seed rack of the Mattson cooker, with equal-weight steel pin plungers positioned on top of each seed so that the needles of the plungers were resting on the seeds. The rack was submerged into boiling water on a hotplate (temperature 95 °C at boiling point at 1,300 m elevation of the laboratory in Kawanda, Uganda). As the seed coat softened during cooking, the plungers pierced the cooked seeds and the drop time was recorded when the steel pin made contact with the metal plate. CKT (min) was recorded as the time elapsed from immersion in hot water until the 20th steel pin dropped (Wang and Daun 2005). Six, four, eight, and six cookers (machine) were used during the process in cycles 1A, 1B, 2A and 2B, respectively. For measurement of Fe and Zn content, cooked seeds were oven-dried at 60 ºC for at least 12h and ground into a powder using a Sunbeam conical burr coffee grinder EM0480 (Sunbeam, Australia). The concentration of Fe and Zn (mg kg^−1^) was determined using EDXRF spectrometry (Paltridge et al. 2012).

### Relationship matrices

Three relationship matrices were constructed: (i) a pedigree-based relationship matrix (A-matrix) (Henderson 1976), (ii) genomic relationship matrix (G-matrix) based on whole-genome SNP data (VanRaden 2008), and (iii) a hybrid matrix (H-matrix) combining pedigree and genomic relationship information (Legarra et al. 2009).

SNP data from four cycles (Cycle 1A, 1B, 2A and 2B) were combined and filtered to remove SNP markers with minor allele frequency < 0.01 and with more than 20% missing data. Individuals with more than 20% missing SNP data were also removed. The cleaned SNP data included 3792 individuals and 1791 markers, and the G-matrix (${\boldsymbol{G}}$) was constructed using VanRaden method I (VanRaden 2008) in R package ASRgenomics (Gezan et al. 2025) as shown in Eq. 2.

2 $${\boldsymbol{G}}=\frac{\left({\boldsymbol{M}}-{\boldsymbol{P}}\right)({\boldsymbol{M}}-{{\boldsymbol{P}})}^{\boldsymbol{^{\prime}}}}{2\sum_{{\boldsymbol{i}}=1}^{{\boldsymbol{m}}}{{\boldsymbol{p}}}_{{\boldsymbol{i}}}(1-{{\boldsymbol{p}}}_{{\boldsymbol{i}}})}$$

where ${\boldsymbol{M}}$ is the allele sharing matrix, with ***n*** rows of individuals and ***m*** columns of markers; ${\boldsymbol{P}}$ is the matrix of allele frequencies, ${{\boldsymbol{p}}}_{{\boldsymbol{i}}}$ is the observed frequency of the ***i***-th marker of all genotyped individuals.

The A-matrix was constructed in ASReml-R 4.2, and the H-matrix was constructed using R package ASRgenomics by combining A- and G-relationship matrices. For individuals with both genomic and pedigree-based relationship information, a scaling parameter (λ = 0.95) was used to weight the differences between genomic and pedigree relationship so that for the subset of individuals with both pedigree and genomic information (${{\boldsymbol{A}}}_{22}$), 95% of the information will be taken from ${\boldsymbol{G}}$ and the remaining 5% from ${{\boldsymbol{A}}}_{22}$. The inverse of H-matrix (Aguilar et al. 2010; Martini et al. 2018) is computed as shown in Eq. 3.

3 $${{\boldsymbol{H}}}^{-1}= {{\boldsymbol{A}}}^{-1}+ \left[\begin{array}{cc}0& 0\\ 0& \lambda ({{\boldsymbol{G}}}^{-1}-{{\boldsymbol{A}}}_{22}^{-1})\end{array}\right]$$

The A- and H-matrices had 5326 individuals with pedigree information while the G-matrix had a subset of these with 3792 genotyped individuals (Table 2, Fig. 3). The A- and H-matrices included 1439 individuals without phenotypes (ancestors, benchmark and local check varieties, founder parents, and progeny) and 176 which were phenotyped but did not have genotypes (Fig. 3, Table 1). In the G-matrix of 3792 individuals, 84 did not have phenotypes (Fig. 3).

**Table 1:** Number of common bean individuals that were genotyped and with or without phenotypes in relationship A-, G- and H-matrices

Individuals	No. individuals in relationship matrices			
	G-matrix	A and H-matrices		
	No. individuals genotyped	No. individuals with phenotypes	No. individuals without phenotypes	Total
Cycle 1A progeny	870	1028	2	1030
Cycle 1B progeny	1038	1034	5	1039
Cycle 2A progeny	905	906	1	907
Cycle 2B progeny	840	851	0	851
Founder parents	113	52	66	118
Ancestors and local checks	26	16	1365	1381
Total	3792	3887	1439	5326

### Population structure based on SNP data

The population structure was assessed by performing a principal component analysis (PCA) of SNP data to visualise the genetic relationships among founder parents, cycle 1 and cycle 2 progeny of populations A and B.

### Multivariate linear mixed model analysis

All five traits (CKT, Fe, Zn, SW100 and WAC) were analysed in a multivariate LMM to derive the best linear unbiased predictions of genetic effects as shown in Eq. 4.

4 $${\boldsymbol{Y}}=\boldsymbol{X}\boldsymbol{B}+{{\boldsymbol{Z}}}_{{\boldsymbol{g}}}{{\boldsymbol{U}}}_{{\boldsymbol{g}}}+{\boldsymbol{E}}$$

where ${\boldsymbol{Y}}$ is the matrix of phenotypes for CKT, Fe, Zn, SW100 and WAC; ${\boldsymbol{B}}$ is the matrix of fixed effects, including machine effect for CKT and overall mean for the traits; ${\boldsymbol{X}}$ is a design matrix of fixed effects; ${{\boldsymbol{Z}}}_{{\boldsymbol{g}}}$ is a design matrix relating phenotypes to genotypes for five traits; ${{\boldsymbol{U}}}_{{\boldsymbol{g}}}$ is the matrix of random genetic effect for five traits with an assumed distribution *N*(0, ***K***$\otimes {\boldsymbol{G}}$), where ${\boldsymbol{G}}$ is the variance–covariance matrices for the random effect for each trait; ***K*** is the relationship matrix used in the model, based on A-, G-, or H-matrix; and ${\boldsymbol{E}}$ is a matrix of random residual effects for the traits following a distribution of *N* (0, ***I***$\otimes {\boldsymbol{R}}$), where ${\boldsymbol{R}}$ is the variance–covariance matrix for the error term. General heterogenous correlation (corgh) and unstructured (us) variance structure were assumed for additive and residual effects, respectively, allowing for modelling of correlation and covariance of these effects.

Breeding values of individuals were the predicted random genetic effects (ABLUPs, GBLUPs or HBLUPs) from the multivariate LMM with the A-, G-, or H-matrices, respectively.

#### *Narrow-sense heritability*

Narrow-sense heritability (${h}^{2}$) for each trait was defined as the ratio of additive variance to the total variance (5) as shown in Eq. 5.

5 $${h}^{2}=\frac{{\sigma}_{a}^{2}}{{\sigma}_{a}^{2}+{\sigma}_{e}^{2}}$$

where ${\sigma}_{a}^{2}$ is the additive variance and ${\sigma}_{e}^{2}$ is the residual variance component from each BLUP model. This definition of heritability is subject to the limitations of the corresponding BLUP models as the ability to capture additive genetic effects varies across relationship matrices. For example, additive variance captured by the genomic model depends on the linkage disequilibrium between markers and quantitative trait loci (QTL), and ${h}^{2}$ from models based on the G-matrix is described as genomic heritability (de los Campos et al. 2015).

#### *Accuracy of breeding values based on predicted error variance*

Prediction error variance (PEV)-based accuracy of breeding values ($r$) for each individual ($i$) from each model is calculated as shown in Eq. 6.

6 $${r}_{i}=\sqrt{1-\frac{{PEV}_{i}}{{{\boldsymbol{K}}}_{ii}{\sigma}_{a}^{2}}}$$

where ${{\boldsymbol{r}}}_{{\boldsymbol{i}}}$ is the PEV-based accuracy of the breeding value for individual ${\boldsymbol{i}}$, ${PEV}_{i}$ is the prediction error variance for individual ${\boldsymbol{i}}$, ${{\boldsymbol{K}}}_{ii}$ is the diagonal element of the relationship matrix ***K*** used in the model corresponding to the individual ${\boldsymbol{i}},$ and ${{\boldsymbol{\sigma}}}_{{\boldsymbol{a}}}^{2}$ is the additive genetic variance of the trait estimated from the model (Gilmour et al. 2022). The number of individuals in A- and H-matrices was greater than the number in the G-matrix (Table 1). Therefore, the accuracy of breeding values across LMMs was assessed using the subset of genotypes common to the A-, G- or H-matrices.

#### *Correlation of additive genetic and residual effects among the traits*

Multivariate LMM allowed estimation of the correlation of additive genetic and residual effects across traits through the assumed covariance structures. A ‘corgh’ variance structure was assumed for the additive effects and ‘us’ variance structure for the residual effects (Butler et al. 2023). Variance–covariance matrices for the residual effects were extracted from the model, and pairwise correlations of residuals among traits are estimated as shown in Eq. 7.

7 $${\rho}_{xy} = \frac{{\sigma}_{xy}}{\sqrt{{{\sigma }^{2}}_{x}* {{\sigma }^{2}}_{y}}}$$

where ${\rho}_{\mathrm{x}\mathrm{y}}$ is the correlation coefficient between the residual effects of traits $x$ and $y$, ${\sigma}_{\mathrm{x}\mathrm{y}}$ is the covariance between the residual effects of traits $x$ and $y$, ${{\sigma }^{2}}_{x}$ and ${{\sigma }^{2}}_{y}$ are the residual variances of traits $x$ and $y$, respectively.

### Genetic gain across cycles

Genetic gain for each trait across cycles 1 and 2 in populations A and B was evaluated by measuring the change in the mean trait values from cycle 1 to cycle 2 within each population. Trait values (ABLUPs, GBLUPs or HBLUPs plus the overall mean for each trait) were averaged separately over cycles 1 and 2 in populations A and B. For example, the realised genetic gain in population A from cycle 1 to cycle 2 was defined as the unit change in average trait values from cycle 1A to cycle 2A and was expressed as a percentage of cycle 1A average trait values. Since the cycle length was 2 years, annual genetic gain was half of these values.

To account for the shrinkage commonly associated with the BLUP estimates (Holland and Piepho 2024), realised genetic gain was also calculated using deregressed BLUPs (Garrick et al. 2009). Deregressed BLUPs (dBLUPs) are obtained as shown in Eq. 8.

8 $${\mathrm{d}\mathrm{B}\mathrm{L}\mathrm{U}\mathrm{P}}_{i} = \frac{{\mathrm{B}\mathrm{L}\mathrm{U}\mathrm{P}}_{i}}{{r}_{i}^{2}}$$

### Predictive ability and rank correlations across population, cycles and traits

Predictive ability was evaluated across populations, across and within cycles and traits by retaining the phenotypic data for the training (reference) population in the analysis while masking the phenotypic data for the validation population. Predictive ability was defined as the Pearson correlation between the BLUPs for the random genetic effects of the validation population and their observed phenotypes (Diaz et al. 2021; Hayes et al. 2021).

Six scenarios were evaluated (Table 2). In the first scenario, phenotypic data from cycle 1A (training population) were used to predict breeding values (BLUPs of random genetic effects) of cycle 2A progeny (validation population). The second scenario predicted breeding values for cycle 2B progeny based on cycle 1B as the training population. In the third and fourth scenarios, combined data from both cycle 1A and 1B were used to predict breeding values in cycle 2A and 2B progeny, respectively. In scenario five, phenotypic data from cycle 1A, 1B and 2A were used to predict breeding values in cycle 2B progeny.

**Table 2:** Scenarios for evaluating predictive ability of multivariate linear mixed models for laboratory traits in common bean based on pedigree, genomic or combined relationship matrices

Scenarios	Training population	Validation population	SNP/pedigree				Phenotypic data			
			1A	1B	2A	2B	1A	1B	2A	2B
1. 2A predicted from 1A	1A	2A	✓	x	✓	x	✓	x	x	x
2. 2B predicted from 1B	1B	2B	x	✓	x	✓	x	✓	x	x
3. 2A predicted from 1A and 1B	1A, 1B	2A	✓	✓	✓	x	✓	✓	x	x
4. 2B predicted from 1A and 1B	1A, 1B	2B	✓	✓	x	✓	✓	✓	x	x
5. 2B predicted from 1A,1B and 2A	1A, 1B, 2A	2B	✓	✓	✓	✓	✓	✓	✓	x
6. 2B single trait prediction	1A, 1B, 2A, 2B	2B*	✓	✓	✓	✓	✓	✓	✓	4

Training and validation populations were defined as population 1A, 1B, 2A and 2B. SNP and pedigree data were available for both training and validation populations, while phenotypic data were restricted to the training populations. '✓' = data on five traits used in the LMM; 'x' = data not used in LMM; '4' = phenotypic data for one trait were masked while data on the four remaining traits were retained

* In scenario 6, predicted breeding values of a trait were based on the four remaining traits in cycle 2B in a leave-one-trait-out approach

In the sixth scenario, we employed a within-cycle correlated trait prediction using leave-one-trait-out approach, wherein a single trait of cycle 2B progeny was predicted from a model trained with all cycle 1A, 1B, 2A and 2B data, by masking its phenotypic records while retaining the records for the other four traits in cycle 2B. For example, when predicting CKT in cycle 2B progeny, their CKT observations were excluded from the model, but observations for Fe, Zn, SW100 and WAC from cycle 2B, along with all available data from other cycles, were retained in the analysis. This scenario allowed us to evaluate the potential of multivariate analysis to predict a difficult-to-measure trait such as CKT based on easier-to-measure, genetically correlated traits such as SW100 and WAC.

Breeding values for each scenario were predicted in multivariate LMM with A-, G- or H-matrices. The individual ranks of cycle 2B population based on their BLUPs from scenarios five and six (predicted breeding values) were compared to their ranks when their phenotype data were included in the model (realised breeding values). The ranks based on predicted values and realised values were compared by a Spearman rank correlation following methods outlined in Simiqueli et al. (2023).

## Results

### Whole-genome SNP data summary across cycles

The 1861 whole-genome SNP markers in the common bean mid-density panel (Ariza-Suarez et al. 2023) gave consistent calls for homozygous and heterozygous SNP loci for individuals such as founder parents and benchmark varieties within and across years. Where slight discrepancies in SNP alleles occurred in replicate genotyping of these varieties, we chose the replicate with the least SNP missing values to include in the relationship matrix. Heterozygosity at SNP loci in the majority of founder parents was generally low (median 1.0%), which reflects the expected near-homozygous status of advanced lines and released varieties in the African bean panel (Saradadevi et al. 2021). However, a few founder parents were moderately to highly heterozygous with heterozygosity up to 65% at SNP loci in one founder line (Online Resource 1).

In contrast, the median value of heterozygosity in cycle 1 F_2_ individuals in cycle 1 was 20.8%, and in S_0_ individuals (progeny of F_1_ x F_1_ crosses) was 31.4%. The median value of heterozygosity in S_0_ individuals in cycle 2 was 21.8%. This contrasts with the self-progeny of parent plants of cycle 2 (S_**2**_, F_**4**_ or higher levels of selfing) which averaged 5.8% median heterozygosity.

High levels of heterozygosity were achieved in cross progeny in both cycles 1 and 2, which reflects the choice of genetically distinct pairs of cross parents by OCS implemented in MateSel.

### Correction of pedigrees with whole-genome SNP data

Some founder parents shared similar whole-genome SNP profiles to other founder parents; for example, SNP data showed that bean variety PERRYMARROW was closely related to AND277, and Masindi yellow long was closely related to Amendoin and DAB366. In total, 14 founder parents had SNP data that were similar to one or more other founder parents. All named founder parents with SNP genotypes were included in the genomic relationship matrix.

Among the initial 2546 cycle 1 (combined populations A and B) putative S_0_ and F_2_ progeny, 383 were identified as inadvertent selfs of one of the maternal founder parents and 57 as inadvertent selfs of the maternal F_1_ parent, that is, they were F_2_ individuals. The pedigree of inadvertent selfs was corrected to match their parentage based on whole-genome SNP data. Moreover, some genotyped plants either died after genotyping or produced few or no S_0,1_ seeds, leaving 2128 cycle 1 individuals (including confirmed S_0_ cross-progeny and intended or inadvertent selfs of parent plants) of which 2062 were evaluated in the laboratory for quality traits.

In cycle 2 (combined populations A and B), the SNP genotypes of 1691 putative S_0_ progeny were compared to the SNP genotypes of their cycle 1 S_1_ parent plants (as interpreted from the self-progeny of these parent plants), and 356 individuals carried unique SNP alleles from the maternal plant and none from the paternal plant. These were classified as inadvertent selfs of cycle 1 maternal plants and their pedigree was corrected to match their parentage based on whole-genome SNP data. In cycle 2, 1757 individuals (including true S_0_ progeny and intended and inadvertent selfs) produced sufficient self-bulk seeds to proceed for phenotyping.

In summary, 15–20% putative S_0_ progeny plants in cycles 1 and 2 were identified as inadvertent selfs of maternal plants through whole-genome SNP genotyping, and their pedigree was corrected. These individuals continued in the evaluation programme as selfs of the maternal parent.

### Population structure

Among the 91 founder parents chosen for crossing in population A and 88 founder parents in population B, 21 were common between populations A and B, and therefore, 158 unique founder parents were used to begin cycle 1 across populations A and B (Online Resource 1).

**Fig. 4:**
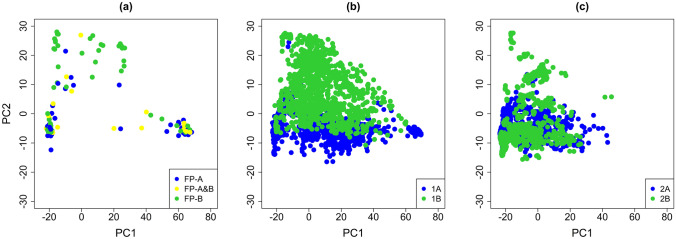
Biplots of principal component analysis based on 1791 SNP markers in the common bean mid-density panel showing **a** the distribution of founder parents in population A (‘FP-A’, blue) and population B (‘FP-B’, green) and founder parents used in both Founder A and B (‘FP-A&B’, yellow), **b** cycle 1 progenies of population A (‘1A’, blue) or population B (‘1B’, green), and **c** cycle 2 progenies of population A (‘2A’, blue) or population B (‘2B’, green). Each point represents the location of an individual in the two-dimensional array defined by the eigen vectors of the first (PC1) and second (PC2) principal components explaining 22% and 5% of total variance, respectively

Founder parents in populations A and B originated in the Mesoamerican and Andean gene pools (Saradadevi et al. 2021). The founder parents and progeny from cycles 1A and 1B formed two overlapping but apparently separated clusters in two-dimensional arrays (Fig. 4a, b) and three-dimensional arrays (Online Resource 2), and progeny from cycles 2A and 2B showed a higher degree of overlap, particularly in the central region of the PCA plot, suggesting increased genetic admixture among populations A and B in cycle 2 (Fig. 4c, Online Resource 2).

### Mean parental coancestry

Mean parental coancestry among founder parents selected in the optimised crossing list in OCS to begin cycle 1 was 0.075 and 0.032 for populations A and B, respectively. Mean parental coancestry of cycle 1 progeny selected as parents in the optimised crossing list in OCS to begin cycle 2 was 0.120 and 0.050 for populations A and B, respectively.

### Narrow-sense heritability

Estimates of ${h}^{2}$ **(**Table 3**)** from the multivariate LMM across years were lower in the genomic model (average 0.34) than in the pedigree model (average 0.47). However, the ${h}^{2}$ estimates in the single-step model based on the H-matrix (average 0.57) were higher than in both the pedigree and genomic models, except for CKT. This suggests that more of the total variance is explained by additive variance in the single-step model compared to the pedigree and genomic models for Fe, Zn, SW100 and WAC (Online Resource 3). CKT had the lowest ${h}^{2}$ of all traits in the genomic model (0.16), but this was higher in the single-step model (0.43).

**Table 3:** Narrow-sense heritability (${h}^{2}$) ± standard error (se) for time taken to cook 20 beans (CKT) in a sample of 25 beans, iron (Fe) and zinc (Zn) content in the dried and powdered cooked seeds, weight of 100 seeds (SW100) and water absorption capacity (WAC) from multivariate LMMs based on pedigree (A), genomic (G) and combined pedigree and genomic (H) relationship information

Trait	Pedigree (A)		Genomic (G)		Combined (H)	
	${h}^{2}$	se	${h}^{2}$	se	${h}^{2}$	se
CKT	0.49	0.03	0.16	0.02	0.43	0.03
Fe	0.33	0.03	0.31	0.03	0.54	0.03
Zn	0.40	0.03	0.35	0.03	0.55	0.03
SW100	0.56	0.02	0.49	0.03	0.71	0.02
WAC	0.58	0.02	0.39	0.03	0.62	0.02
Average	0.47		0.34		0.57	

### PEV-based accuracy of breeding values

**Fig. 5:**
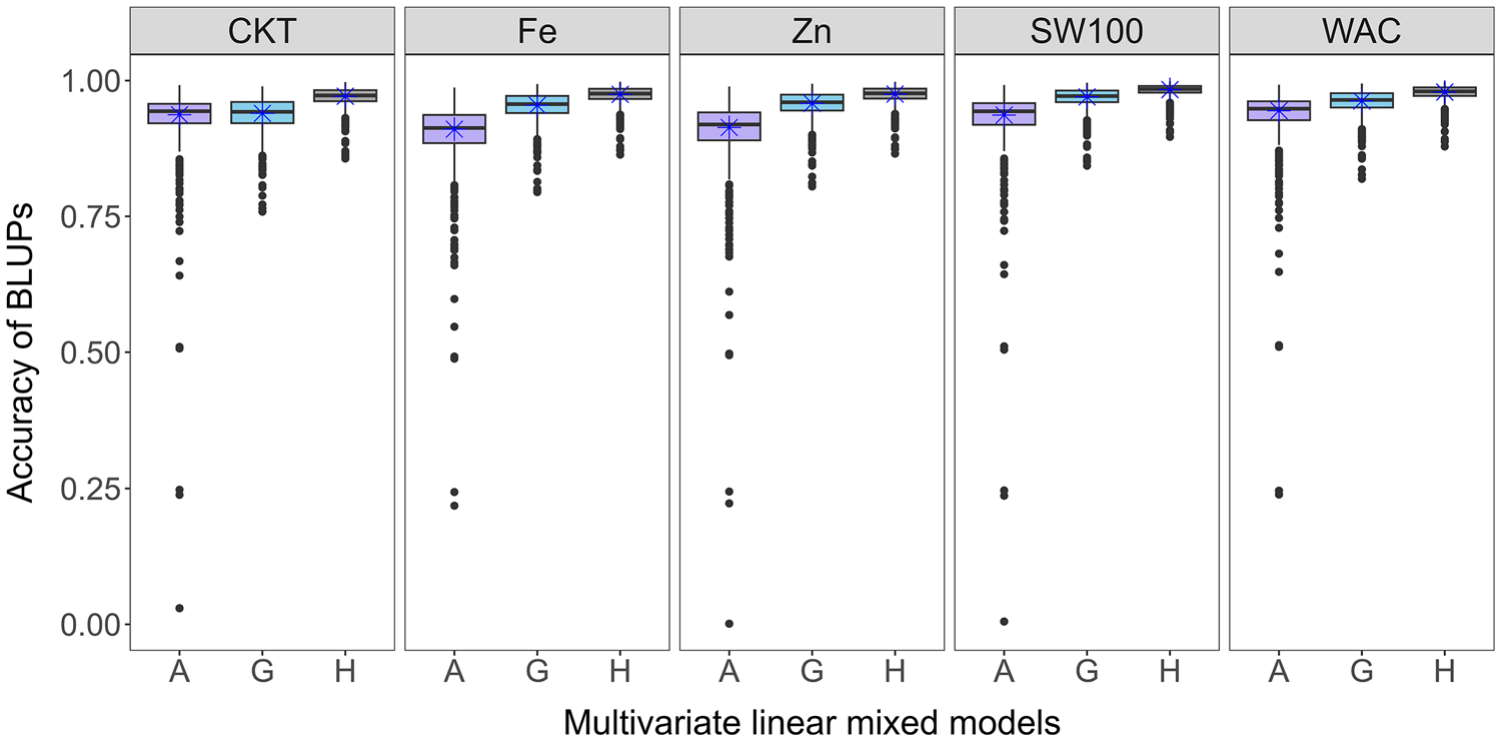
Box plots of PEV-based accuracies of best linear unbiased predictions (BLUPs) of 3752 genotyped individuals of common bean for cooking time (CKT), seed iron content (Fe), seed zinc content (Zn), 100-seed weight (SW100) and water absorption capacity (WAC) from multivariate LMM with A-, G- and H-matrices. The boxes represent the interquartile range and contain 50% of the values, the vertical lines represent the upper and lower quartiles, the horizontal line represents the median, the blue asterisk represents the mean, and dots represent outliers

Average PEV-based accuracy of breeding values (BLUPs) of the 3792 genotyped individuals across all traits and years was high (> 0.9) in the multivariate LMM. PEV-based accuracy was lowest in models based on the A-matrix and highest in models based on the H-matrix (Fig. 5). The PEV-based accuracy of HBLUPs was on an average 5% higher than ABLUPs and 2% higher than GBLUPs. Additionally, HBLUPs across all traits had fewer outliers with low accuracy values compared to ABLUPs and GBLUPs (Fig. 5). The mean PEV-based accuracy of HBLUPs in 2,535 S0 individuals in cycles 1 and 2 was: CKT (0.974), Fe (0.977), Zn (0.978), SW100 (0.985), WAC (0.981).

### Realised genetic gain from cycle 1 to cycle 2

**Fig. 6:**
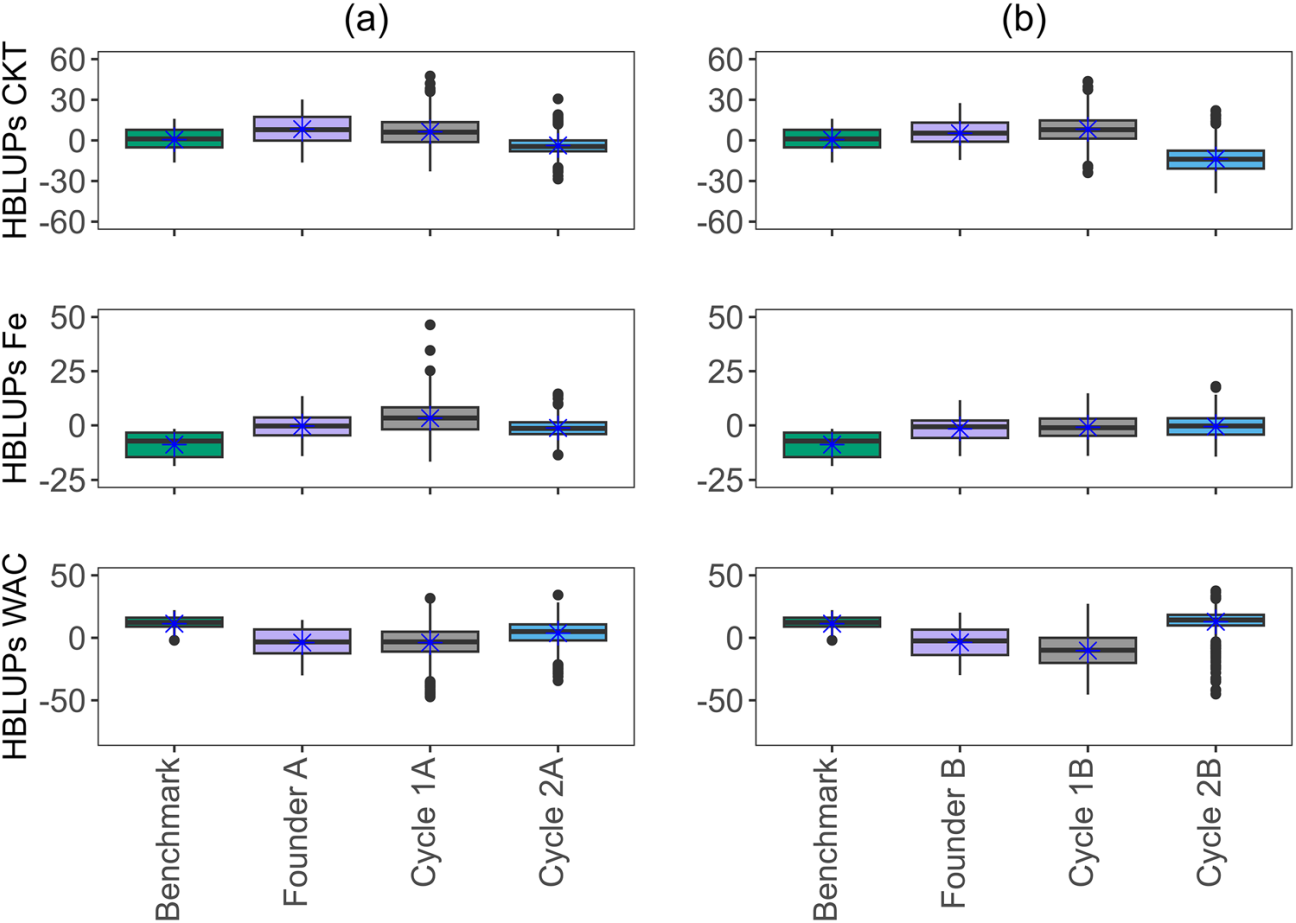
Box plots of HBLUPs for cooking time (CKT; upper panel), seed iron (Fe; middle panel) and water absorption capacity (WAC; lower panel) in 14 common bean benchmark varieties, and in founder parents, cycle 1 and cycle 2 progeny of **a** population A and **b** population B, from multivariate LMM with the combined pedigree and genomic (H) relationship matrix. In each box plot, the boxes represent the interquartile range and contain 50% of the values, the vertical lines represent the upper and lower quartiles, the horizontal line represents the median, the blue asterisk represents the mean, and dots represent outliers

Genetic gain was calculated separately for populations A and B to assess within-population response to selection. Realised genetic gain estimates based on BLUPs (Table 4) were comparable to those obtained using dBLUPs (Online Resource 4). Multivariate LMM with the G-matrix (genomic model) provided more conservative estimates of genetic gain, while the A-matrix (pedigree model) yielded consistently higher and possibly inflated estimates of genetic gain, especially for traits such as CKT and WAC (Table 4). Single-step model generally provided an intermediate estimate of genetic gain (Fig. 6, Table 4).

**Table 4:** Genetic gain in common bean breeding populations A and B for laboratory traits in three multivariate LMM models based on pedigree relationship information to generate ABLUPs, genomic relationship information to generate GBLUPs and single-step genomic analysis to generate HBLUPs

Model	Laboratory trait (units)	Mean trait values							Genetic gain		*p*-value (t-test)	
		1A	1B	2A	2B	FP1A	FP1B	BM	2A|1A	2B|1B	2A|1A	2B|1B
Pedigree	CKT20 (min)	65.50	68.30	51.28	38.67	69.09	66.95	54.15	− 14.22 (− 22%)	− 29.63 (− 43%)	0.00	0.00
	Fe (ppm)	62.00	57.70	57.34	57.92	59.92	57.97	49.06	− 4.66 (− 8%)	+ 0.22 (+ 0%)	0.00	0.34
	Zn (ppm)	25.45	25.43	24.12	24.33	25.05	25.35	20.92	− 1.33 (− 5%)	− 1.10 (− 4%)	0.00	0.00
	SW100 (g)	25.72	27.01	23.46	24.98	27.65	26.75	32.13	− 2.26 (− 9%)	2.03 (− 8%)	0.00	0.00
	WAC (%)	90.49	83.72	98.67	110.33	87.69	86.77	108.38	+ 8.18 (+ 9%)	+ 26.61 (+ 32%)	0.00	0.00
Genomic	CKT20 (min)	71.03	72.37	62.17	54.14	73.40	69.20	67.05	− 8.86 (− 12%)	− 18.23 (− 25%)	0.00	0.00
	Fe (ppm)	60.61	57.23	57.00	57.70	57.03	56.07	49.58	− 3.61 (− 6%)	+ 0.47 (+ 1%)	0.00	0.06
	Zn (ppm)	25.13	25.36	24.14	24.37	23.89	24.37	21.07	− 0.99 (− 4%)	− 0.99 (− 4%)	0.00	0.00
	SW100 (g)	28.21	29.12	25.53	26.93	30.20	29.35	34.05	− 2.68 (− 10%)	− 2.19 (− 8%)	0.00	0.00
	WAC (%)	86.81	80.10	93.68	102.04	85.97	87.55	100.19	+ 6.87 (+ 8%)	+ 21.94 (+ 27%)	0.00	0.00
Single-step	CKT20 (min)	71.92	73.73	61.79	51.80	73.84	70.79	66.09	− 10.13 (− 14%)	− 21.93 (− 30%)	0.00	0.00
	Fe (ppm)	61.33	57.13	56.78	57.42	57.69	56.40	49.11	− 4.55 (− 7%)	+ 0.29 (+ 1%)	0.00	0.25
	Zn (ppm)	25.31	25.31	24.08	24.31	24.23	24.59	20.99	− 1.23 (− 5%)	− 1.00 (-4%)	0.00	0.00
	SW100 (g)	27.99	29.09	25.55	26.99	30.08	29.19	34.17	− 2.44 (− 9%)	− 2.10 (− 7%)	0.00	0.00
	WAC (%)	86.18	79.77	93.71	102.88	86.18	86.38	101.32	+ 7.53 (+ 9%)	+ 23.11 (+ 29%)	0.00	0.00

Mean trait values are the mean ABLUPs, GBLUPs or HBLUPs of individuals in populations 1A, 1B, 2A and 2B, founder parents of population 1A (FP1A), founder parents of population 1B (FP1B) or benchmark varieties (BM) plus the population mean (See Online Resource 4 for genetic gain based on deregressed BLUPs). Genetic gain is calculated as the change in mean trait values from cycle 1 to cycle 2 in units of the trait and expressed as a percentage of the cycle 1 mean (in parentheses). The significance of the change in mean trait values from cycle 1 to cycle 2 was expressed as the probability (*p*-value) of a t-test.

Response to selection was strongest for CKT. The mean HBLUPs for CKT decreased significantly from cycle 1 to cycle 2 progeny by 10.1 min in population A, and by 21.9 min in population B (Fig. 6), for an average of 8 min per year or 11% per year reduction in CKT from cycle 1 to cycle 2 in populations A and B in the single-step model (Table 4).

Despite positive weightings for these traits in the selection index, Fe and Zn did not respond positively to selection from cycle 1 to cycle 2. Mean progeny HBLUPs for Fe decreased from cycle 1 to cycle 2 by 4.6 ppm in population A (7% of the population mean) but increased slightly by 0.3 ppm in population B (1% of the population mean) (Fig. 6, Table 4). Similarly, Zn decreased on average by 5% from cycle 1 to cycle 2 in population A, and 4% in population B (Table 4).

WAC responded positively to selection from cycle 1 to cycle 2 in both populations A and B. The mean progeny HBLUPs for WAC increased from cycle 1 to cycle 2 by 7.5% in population A (9% of the population mean), and by 23.1% in population B (29% of the population mean) (Fig. 6, Table 4).

SW100 did not respond to selection for seed weight from cycle 1 to cycle 2. The mean progeny HBLUPs for SW100 decreased from cycle 1 to cycle 2 by 7% to 9% of the population mean in populations A and B in the single-step model (Table 4). This occurred despite positive weighting for higher seed weight in the selection index.

### Additive genetic correlations among traits in multivariate LMMs

**Fig. 7:**
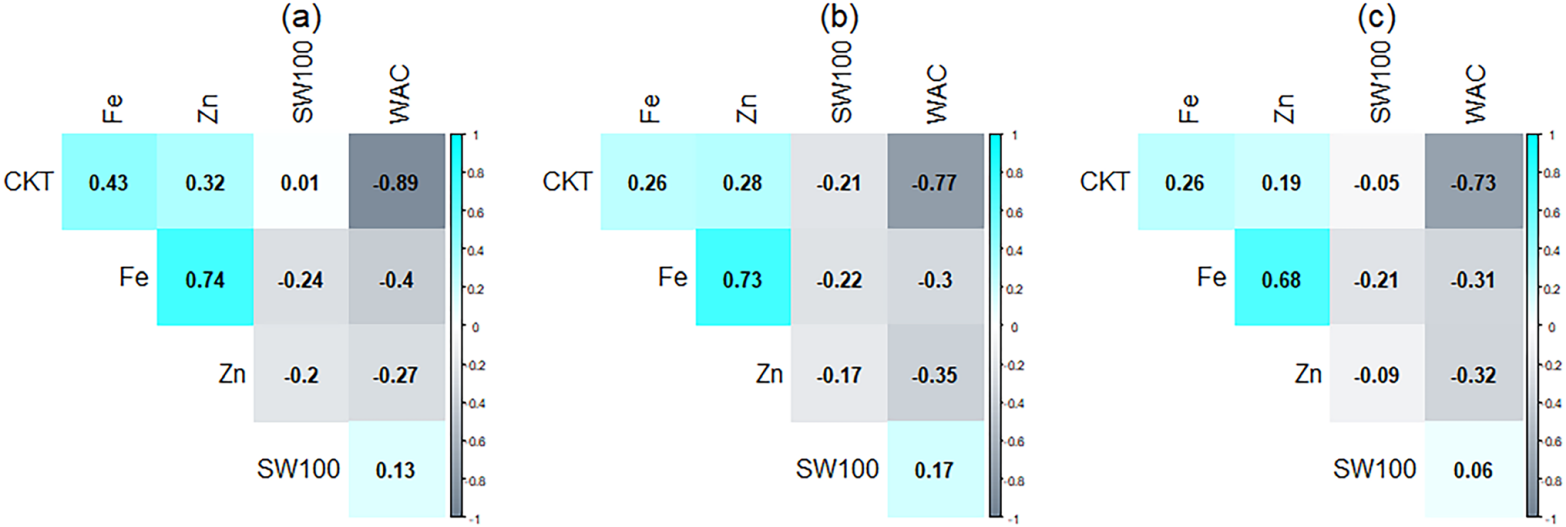
Additive genetic correlations among traits from multivariate LMMs using **a** pedigree, **b** genomic and **c** a combination of pedigree and genomic relationship information (single-step model). Correlations greater than 0.10 are significant at *p* < 0.05 according to the Z-ratio test (see Online Resource 5)

Additive genetic correlations among traits in the multivariate LMMs with the A-, G- and H-matrices were similar in pattern but varied in strength of association (Fig. 7). Correlations with the pedigree model (A-matrix) showed greater extremes than in the genomic (G-matrix) or single-step (H-matrix) models, and the single-step model tended to have more conservative values than the genomic model (Fig. 7).

Based on the single-step model, the favourable additive genetic correlations for desired gains were between Fe and Zn (+ 0.68), that is, individuals with higher Fe tended to have higher Zn, and between WAC and CKT (-0.73), that is, individuals with lower CKT tended to have higher WAC.

Several detrimental additive genetic correlations were also observed. In the single-step model, CKT was positively correlated with Fe (+ 0.26) and Zn (+ 0.19), that is, individuals with lower CKT tended to have lower Fe and Zn content. Similarly, SW100 was negatively correlated with Fe (− 0.21) and Zn (− 0.09), meaning that higher Fe or Zn tended to be found in small-seeded beans.

The net result of these correlations from cycle 1 to cycle 2 was a negative response to selection for Fe, Zn or SW100, and genetic gain did not follow the positive weightings in the index for these traits in both populations A and B.

The correlations of residual effects between some pairs of traits were also significant (Online Resource 6). A positive correlation of residual effects of + 0.69 in single-step analysis occurred with the measurement of Fe and Zn in the same samples on the EDXRF machine. Some samples were subject to substantial machine or sample error variance, but other samples were not, and the pattern was the same for both Fe and Zn. A small but significant negative correlation of residual effects was observed for CKT *vs* WAC. These traits were measured either prior to (WAC) or after cooking (CKT) on the same biological samples. Samples with high residual error for CKT had low residual error for WAC. Complex interactions between samples (genotypes) and machines in the laboratory may contribute to these residual correlations among traits.

### Forward predictive ability

Predictive ability, defined as the correlation between observed phenotypic values (recorded in the laboratory) and predicted breeding values (BLUPs) in candidates prior to phenotyping was estimated for the multivariate LMM with the A-, G- and H-matrices, under six prediction scenarios across cycles and two populations (Table 2). In each scenario, the training data were analysed in LMM with the pedigree, genomic or single-step model, and BLUPs from the model were correlated with the observed values of the validation set.

Forward predictive ability (scenarios one to five) was highest for SW100 (population A > 0.5 and population B > 0.3), intermediate for Fe and WAC (0.2–0.3) and lowest for CKT and Zn (< 0.1) (Table 5).

**Table 5:** Forward predictive ability measured as Pearson correlation coefficients between predicted breeding values from the training population and observed values in the validation population (phenotypic values recorded in the laboratory) of common bean across two cycles of selection (cycles 1 and 2) in two breeding populations (populations A and B). Breeding values were predicted using multivariate linear mixed models (LMM) with pedigree (A-matrix), genomic (G-matrix) or combined (H-matrix) relationship matrices

Prediction scenarios	Matrix used in LMM	Training population	Validation population	CKT20	Fe	Zn	SW100	WAC
1. 2A predicted from 1A	A-matrix	1A	2A	0.10	0.05	− 0.01	0.50	0.05
	G-matrix	1A	2A	0.12	0.10	0.06	0.56	0.10
	H-matrix	1A	2A	0.09	0.09	0.07	0.54	0.10
2. 2B predicted from 1B	A-matrix	1B	2B	− 0.05	0.08	− 0.01	0.30	0.13
	G-matrix	1B	2B	0.06	0.19	0.07	0.45	0.23
	H-matrix	1B	2B	− 0.07	0.19	0.03	0.34	0.18
3. 2A predicted from 1A and 1B	A-matrix	1A + 1B	2A	0.07	0.06	− 0.02	0.52	0.01
	G-matrix	1A + 1B	2A	0.23	0.13	0.04	0.56	0.21
	H-matrix	1A + 1B	2A	0.17	0.13	0.05	0.55	0.19
4. 2B predicted from 1A and 1B	A-matrix	1A + 1B	2B	− 0.03	0.20	0.01	0.41	0.11
	G-matrix	1A + 1B	2B	0.08	0.32	0.04	0.48	0.24
	H-matrix	1A + 1B	2B	0.02	0.32	0.02	0.38	0.22
5. 2B predicted from 1A, 1B and 2A	A-matrix	1A + 1B + 2B	2B	− 0.09	0.16	− 0.03	0.41	0.13
	G-matrix	1A + 1B + 2B	2B	0.04	0.32	0.13	0.52	0.25
	H-matrix	1A + 1B + 2B	2B	0.02	0.30	0.09	0.43	0.24
6. 2B single trait predicted from 2B remaining phenotypes and 1A, 1B and 2A	A-matrix	1A + 1B + 2A + 2B (4 traits)	2B single trait	0.48	0.84	0.41	0.73	0.54
	G-matrix	1A + 1B + 2A + 2B (4 traits)	2B single trait	0.47	0.71	0.42	0.68	0.45
	H-matrix	1A + 1B + 2A + 2B (4 traits)	2B single trait	0.49	0.74	0.45	0.60	0.46

In scenarios one to five, the genomic model provided higher predictive ability than the pedigree-based model, particularly for CKT, SW100 and WAC, where it consistently outperformed both pedigree and single-step models across these prediction scenarios (Table 5). Predictive ability with the single-step model was similar to or slightly less than with the genomic model (Table 5). The results underscore the advantage of genomic-based models in leveraging additional data to enhance predictive ability compared to pedigree models, but also show that most traits had low predictive ability across populations and cycles.

Interestingly, in scenario 6, when only one trait was masked from cycle 2B and phenotypic data for the four remaining traits for cycle 2B were included in the model along with previous cycle data (within-cycle correlated trait prediction in a leave-one-trait-out approach), the predictive ability of all traits across pedigree, genomic and combined models were moderate to high. The highest predictive ability for CKT and Zn was achieved in scenario 6 (Table 5).

### Rank correlation between realised and predicted breeding values

**Fig. 8:**
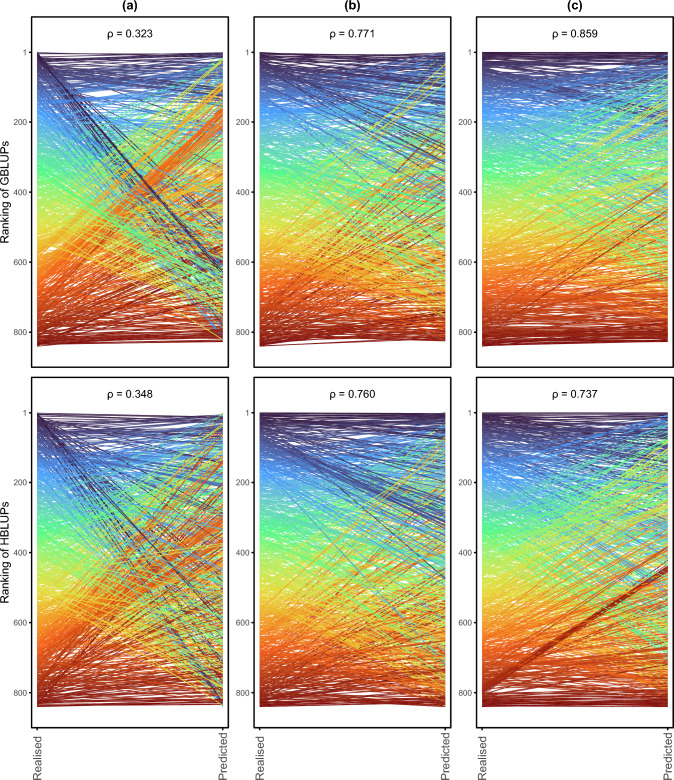
Spearman rank correlation (${\boldsymbol{\rho}}$) between realised and predicted breeding values for cycle 2B progeny, when phenotypic records for all traits in cycle 2B were masked during model fitting, for **a** cooking time, **b** seed iron concentration, and **c** 100-seed weight. Realised GBLUPs (top panel) and HBLUPs (bottom panel) were derived from multivariate linear mixed models fitted with genomic and hybrid relationship matrices, respectively, using all phenotypic data including cycle 2B

**Fig. 9:**
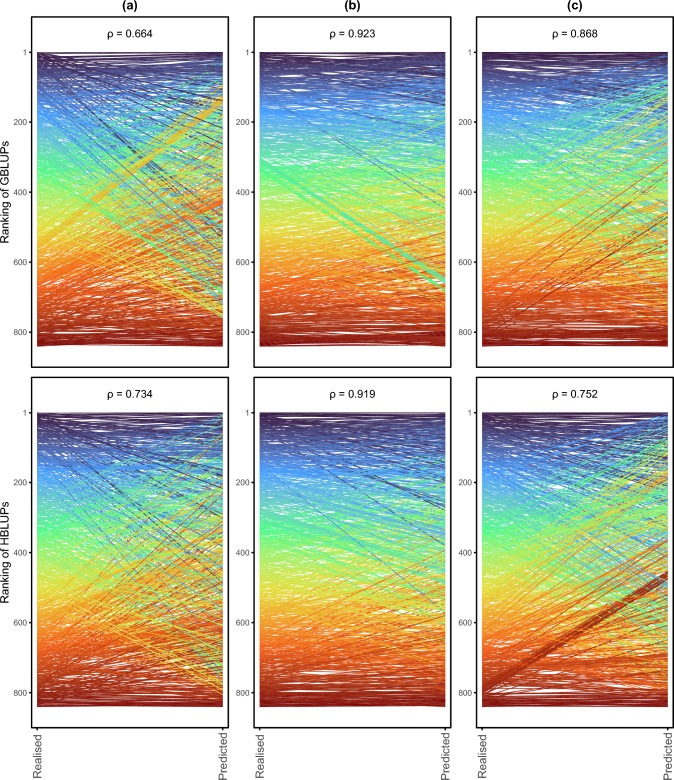
Spearman rank correlation ($\rho$) between realised and predicted breeding values for cycle 2B progeny, when phenotypic records for that trait alone were masked in cycle 2B during model fitting in a leave-one-trait-out approach, for: **a** cooking time, **b** seed iron concentration, and **c** 100-seed weight. Realised GBLUPs (top panel) and HBLUPs (bottom panel) were derived from multivariate linear mixed models fitted with genomic and hybrid relationship matrices, respectively, using all phenotypic data including cycle 2B

Spearman rank correlations between realised and predicted breeding values of cycle 2B under prediction scenario 5 were low for CKT (0.323 and 0.348, respectively, for GBLUPs and HBLUPs), but were moderate to high for Fe and SW100 (Fig. 8). When correlated trait phenotypes were included in the model in a leave-one-trait-out approach (scenario 6), rank correlations increased substantially for all traits, with the largest improvement observed for CKT with 0.664 and 0.734 for GBLUPs and HBLUPs, respectively (Fig. 9).

## Discussion

Rapid genetic gain for CKT was achieved in an African common bean breeding programme for low-to-medium heritability seed traits across short (two-year) cycles of early-generation selection. This is one of the first uses of multivariate single-step genomic selection to evaluate genetic gain across breeding cycles in self-pollinating crops. Each year, the breeding values of all available individuals were submitted to OCS based on an index of multiple traits to generate optimised crossing lists. This breeding approach has been broadly validated in tree breeding (El-Kassaby et al. 2024, Isik and McKeand 2019; Isik et al. 2026) and in livestock breeding, where OCS based on an index of multiple traits is the gold standard for promoting genetic gain while conserving genetic diversity (Pook et al. 2026).

We found unexpected detrimental and beneficial genetic correlations among traits during two cycles of selection with OCS, and were able to make timely adjustments to the weights on Fe and Zn in the index to generate an optimised crossing list to begin cycle 2 in population B. This resulted in higher Fe but not Zn in population B cycle 2 progeny (Table 4), and it is clear that further adjustments will be required to the economic weights on Fe and Zn in future cycles. There were also laboratory and sample-related sources of variation which contributed to residual correlations among traits, especially in the measurement of Fe and Zn on the EDXRF machine. This suggests that the measurement of Fe and Zn in some samples is less reliable than in others, based on complex interactions between samples and machines.

This breeding method exploits relationships between individuals within and across breeding cycles, such as between full-sibs and cross-and-self-sibs of S_1_ parent plants (Cowling et al. 2023), and the accuracy of breeding values is expected to increase when the number of related individuals increases (Simm 1998). Correction of pedigrees in 15–20% inadvertent selfs in progeny of cycles 1 and 2 contributed to the high accuracy of HBLUPs in the single-step models. High PEV-based accuracy ($r$ > 0.8) was achieved in S_0_ individuals in previous studies based on this breeding method (Castro-Urrea et al. 2023; Cowling et al. 2023). We observed very high PEV-based accuracy of breeding values which increased from A-, G- to H-matrices (Fig. 5). This could explain why there were only minor differences between genetic gain estimates derived from BLUPs and dBLUPs. In this dataset, most progeny were phenotyped and individuals were well connected through genomic and/or pedigree information, resulting in high reliability and limited shrinkage effects (Holland and Piepho 2024).

The high PEV-based accuracy of breeding values we observed in CKT occurred despite low genomic heritability for this trait (Table 3). Phenotyping for CKT is particularly time-consuming and labour-intensive, and it takes at least 40 working days to assess CKT on 1,000 individuals over 8 machines. Replication of individuals is impractical due to limited self-seed per plant and the time-consuming nature of this laboratory procedure. Partial replication of designs for CKT based on founder parents or benchmark varieties may help to control various sources of error such as machine or day effects and improve genomic heritability. Our goal is to improve methodology, increase heritability, increase forward prediction ability and potentially reduce the number and cost of CKT measurements in the laboratory.

Single-step genomic analysis with the H-matrix revealed rapid genetic gain for CKT (average -8.0 min *y*^*−*1^ or − 11% population mean *y*^−1^) in this African bean breeding programme. The relatively small increase in mean parental coancestry from cycle 1 to cycle 2 (from 0.032 to 0.050 in population B) confirms that the optimised crossing list based on OCS implemented in MateSel effectively balanced genetic gain while retaining genetic diversity in these populations.

### Multivariate LMM reveals important genetic correlations among seed traits

A major advantage of multivariate LMM is the accurate estimation of genetic correlations among traits, which in turn improves accuracy of breeding values and also may limit simultaneous genetic gain in all traits (Endelman 2023). Multivariate genomic analysis has been used in animal, tree and crop breeding where it has revealed genetic correlations among traits which affect genetic gain in all traits (Calus and Veerkamp 2011; Hayatgheibi et al. 2025).

In our study, significant genetic gain was achieved in the desired negative direction (i.e. reduction) for CKT across both populations A and B. Cycle 2 progeny cooked on average 16 min faster than cycle 1 progeny in single-step genomic analysis, averaged across populations A and B (Table 4). This high genetic gain in CKT supports the results of other studies which revealed significant genetic variability for CKT in common bean (Bassett et al. 2021; Jeffery et al. 2025; Saradadevi et al. 2021). Selection for CKT was effective in this study despite the low heritability of the trait and detrimental correlations with seed Fe and Zn content, possibly because of favourable genetic correlations with WAC (Fig. 7).

At the end of cycle 2, desired genetic gains in Fe and Zn concentrations were not achieved, despite weighting to favour these traits in the selection index. Seed Fe and Zn concentrations were positively correlated with CKT (Fig. 7), which makes it difficult to reduce CKT while increasing Fe and Zn. Likewise, Fe was negatively correlated with SW100 (Fig. 7), and SW100 decreased from cycle 1 to cycle 2 despite weighting to favour SW100 in the selection index. Such trade-offs between traits are well documented in plant breeding, where pleiotropy or linkage disequilibrium constrains balanced progress across traits (Chen and Lübberstedt 2010; Neyhart et al. 2019; Thompson and Meyer 1986). The fact that progress was achieved in CKT but not in Fe and Zn underscores the importance of multivariate models in capturing genetic correlations among traits in early cycles of the breeding programme, so that steps may be taken to counter these correlations in future cycles.

### Breeding with detrimental genetic correlations

Knowledge of detrimental genetic correlations in cycles 1 and 2, as revealed here in common bean, motivates changes in the selection method to balance future genetic improvement across all traits through the use of various algorithms (Yadav et al. 2025). The weighting assigned to various traits in the selection index can be modified to help achieve desired goals (Brascamp 1984; Kinghorn 2025). The use of OCS implemented in software MateSel permits the breeder to balance genetic gain in traits against increases in parental coancestry to meet various market demands in the breeding programme (Kinghorn and Kinghorn 2025). Other methods such as optimal haplotype stacking (Villiers et al. 2024) could also be used to manage the trade-offs among traits imposed by detrimental genetic correlations.

In this study, we reacted to an early warning that Fe was in detrimental correlation with CKT and adjusted the weightings in the selection index for Fe in crossing to begin cycle 2B. We achieved a slight positive gain in Fe of 1% in population 2B over 1B, in contrast with -7% change in mean Fe in population 2A over 1A (Table 4). In the crossing design to begin cycle 2B, we implemented an end-use profile in MateSel which favoured Fe in the cycle 2B crossing design. This result provides confidence that further gains can be made in increasing Fe while decreasing CKT in future cycles of this bean breeding programme. MateSel has been successfully used in loblolly pine to balance genetic gain and diversity across cycles using constrained mate selection that accounts for multiple traits and inbreeding (Isik and McKeand 2019). Other methods of optimisation in crossing designs are also developed, for example, the use of metaheuristic algorithms for genotype building by strategically assembling desirable haplotypes and preserving diversity (Yadav et al. 2025).

Despite the positive genetic correlation between Fe and Zn (Fig. 7), and positive selection for Zn in the index, Zn did not improve in cycle 2B (Table 4). Although a substantial portion of the genetic loci controlling seed Fe and Zn are shared, Zn may be controlled by specific loci that only affect Zn and not Fe (Diaz et al. 2022; Izquierdo et al. 2018). Therefore, selection for Fe and Zn may not always result in equal gains in both elements. There is typically lower genetic variation available for selection in Zn relative to Fe (Mukamuhirwa et al. 2015; Saradadevi et al. 2021). It will be challenging to increase SW100 due to detrimental correlations with Fe and Zn, but this is important to meet market preferences for medium to large seeds.

These challenging results emphasise the importance of considering trait-specific weights in selection index design and cross-optimisation tools in MateSel. The combination of slightly positive genetic gain in Fe alongside substantial improvement in shorter CKT in population B suggests that targeted index weighting and optimal cross selection may achieve progress in CKT, Fe, Zn and SW100. Phenotyping the correlated traits in each cycle will be important because genetic correlations and heritability of traits are likely to change rapidly across cycles of selection, and these changes depend on factors such as effective population size and strength of selection (Chantepie and Chevin 2020).

### Relative performance of pedigree, genomic and single-step models

Reliable estimates of additive variance components are essential for optimal selection efficiency (Thompson and Meyer 1986). The first step in this study to improve reliability of additive variance estimates was the correction of pedigrees based on marker relationships, when 15–20% of cross-progeny were found to be selfs of parent plants and their pedigrees were corrected after SNP genotyping.

Differences observed in this study among the pedigree, genomic and single-step models can be attributed to the underlying information they exploit. The genomic model captures realised Mendelian sampling variation and within-family genetic differences, which the pedigree model cannot detect (Henderson 1984; Hill and Weir 2011). The genomic model resulted in lower heritability for CKT than the pedigree model (average *h*^2^ = 0.16 in genomic model and 0.49 in pedigree model; Table 3), which aligns with previous reports of conservative variance component estimates and shrinkage of GBLUPs in analysis with genomic matrices compared to ABLUPs with pedigree matrices or HBLUPs with H-matrices (Beaulieu et al. 2022; Beaulieu et al. 2020; de los Campos et al. 2015; Jurcic et al. 2021). This shrinkage arises because GBLUP relies on marker-derived genomic relationships, where genetic variance is captured through linkage disequilibrium between markers and causal loci (Beaulieu et al. 2020). When marker density or genome coverage is insufficient to tag a substantial proportion of genetic variance, or when linkage disequilibrium is weak, heritability estimates and subsequently genetic gain can be reduced (de los Campos et al. 2015). In this study, we used a modest set of 1861 markers to construct the G-matrix. Although the marker density was relatively low, our results suggest that these markers maintained sufficient linkage disequilibrium with the traits of our interest to provide accurate breeding values (*r* > 0.9) (Fig. 5).

Conversely, the A-matrix, based solely on pedigree relationships, produced inflated heritability estimates for traits like CKT (Table 3). This inflation of heritability is a known consequence of pedigree-based models, which often overestimate additive variance because they do not account for Mendelian sampling and recombination effects (Ashraf et al. 2016; Henderson 1984). Additionally, pedigree matrices assume that founders are unrelated; this assumption was clearly violated in this population where SNP data indicated high relatedness among some founder parents (Beaulieu et al. 2022; McLean et al. 2024). We conclude that estimates of additive genetic variance from the pedigree model are biased upwards in this study.

However, when the pedigree matrix was combined with the genomic matrix in the single-step model, there was a synergistic benefit which improved the accuracy of breeding values and narrow-sense heritability for traits in the single-step model (Table 3, Fig. 5). The single-step model captures the realised genetic relationships from markers while also retaining information from pedigree links for individuals lacking genomic data. This blended approach appears advantageous for improving accuracy of breeding values and improving heritability estimates while providing a realistic estimate of genetic gain and predictive ability. This is in consensus with the widely reported benefits of single-step GS over genomic or pedigree-based models in various crop, tree and animal species (Ashraf et al. 2016; Christensen et al. 2012; Gao et al. 2012; Jurcic et al. 2021; Legarra et al. 2014; Sood et al. 2020).

### Forward predictive ability and Spearman rank correlation

High predictive ability of traits across cycles in genomic or single-step genomic evaluation may enable accurate selection of cross-progeny individuals after genotyping but before phenotyping has been completed. This approach has the potential to shorten the breeding cycle and accelerate genetic gain in animal breeding (Koo et al. 2023; Pimentel et al. 2019) and perennial plant breeding (Crain et al. 2020; Hayatgheibi et al. 2025), where breeding values of young animals and juvenile plants were forward-predicted for selection prior to phenotyping. In annual crop plants, predictive ability has been calculated through a cross-validation strategy where the reference and validation set were within the same cycle (Ashraf et al. 2016; Crossa et al. 2010, 2011; Diaz et al. 2021; Pérez-Rodríguez et al. 2017; Sood et al. 2020). Only a limited number of studies have evaluated predictive ability across breeding cycles in self-pollinating annual crops, primarily in wheat (Michel et al. 2016, 2020; Sun et al. 2019).

We evaluated predictive ability (the correlation of predicted breeding values in cycle 2B individuals against the realised phenotypes of those individuals) across and within cycles, and rank correlations of predicted and realised breeding values, for the seed traits CKT, Fe, Zn, SW100 and WAC. We found moderate forward predictive ability for SW100 but very low forward predictive ability for CKT and Zn (Table 5). This aligns with the heritability estimates for these traits, which were highest for SW100 and lowest for CKT (Table 3). A similar relationship between ${h}^{2}$ and forward predictive ability was reported in Norway spruce trees (Hayatgheibi et al. 2025).

Low predictive ability for CKT was expected given the observed low genomic heritability of CKT and potential genotype x environment (G x E) interaction as reported for CKT by others (Garcia et al. 2012; Sadohara et al. 2022). The forward predictive ability for CKT improved slightly as data were added across cycles and by combining data from populations A and B, possibly due to increase in reference population size (Lund et al. 2011). However, no model had high forward predictive ability (> 0.5) for any trait except SW100, but this may change as more data are added in future cycles (Michel et al. 2020).

Spearman rank correlation between realised and predicted breeding values for cycle 2B progeny was also moderate for CKT when all data in cycle 2B were masked (Fig. 8). However, in the leave-one-trait-out approach (Table 5, Fig. 9), CKT achieved a Spearman rank correlation of 0.734 between realised and predicted breeding values, which is sufficient to select parents for crossing when CKT data are not available. This high correlation between realised and predicted breeding values from single-step genomic selection opens up some options for future laboratory testing for CKT and other traits, including reducing the number of individuals undergoing testing for CKT, while potentially shortening selection cycles through early use of parents with high predicted breeding values before phenotypes are available.

The rank correlations between realised and predicted breeding values we report for seed traits in common bean are similar to those reported in a recurrent selection breeding programme in Eucalyptus (Simiqueli et al. 2023). Our findings are consistent with results reported in cows where the inclusion of additional correlated traits markedly improved prediction ability of feed intake (> 100% improvement) across both pedigree and genomic-based models (Pszczola et al. 2013). Similarly, inclusion of secondary traits improved predictive ability for grain yield in wheat (Sun et al. 2019). This reflects the strength of multivariate approaches in capturing shared genetic architecture and borrowing information from correlated easier-to-measure traits to improve prediction of traits that are more resource-intensive to phenotype (Pszczola et al. 2013).

### Substantial genetic gain in cooking time despite low genomic heritability and forward predictive ability

The substantial realised genetic gain observed for CKT in this study, despite low genomic heritability and low forward predictive ability across cycles, can be explained by the interaction of trait architecture and environmental influences. Selection was primarily conducted within cycles using multivariate genomic models, where CKT benefited from its strong genetic correlation with WAC and other correlated traits even when CKT itself had relatively low genomic heritability. In contrast, forward predictive ability across cycles for CKT appeared to be constrained by its low genomic heritability. Strong genetic gain in CKT across cycles was helped by high weight for CKT in the selection index, which may be adjusted in future cycles.

While several studies suggest that genotype × environment (G × E) interaction for CKT is generally limited (Cichy et al. 2019; Katuuramu et al. 2020), CKT is influenced by storage conditions and time of storage (Arruda et al. 2012), which may introduce variation across cycles in this study. In addition, although seeds were produced from single plants grown under screenhouse conditions at Kawanda, Uganda, differences in production timing and associated environmental factors across cycles may have influenced seed development and composition. Moderate G × E effects have been reported for traits such as WAC, Fe, and Zn (Katuuramu et al. 2020, 2021), and variation in these correlated traits may reduce the consistency of multivariate relationships across cycles. This, in turn, can indirectly affect the prediction of CKT. Environmental and laboratory effects such as day and batch were not explicitly modelled in the multivariate framework due to the structure of the available data; however, such effects are implicitly captured within the residual and may contribute to reduced consistency of genetic signals across cycles.

### Implications for annual crop breeding programmes

Our results demonstrate the high value of single-step multivariate genomic analysis in plant breeding, especially in the context of rapid cycles of S_0_-derived family selection, where data are sparse, nonreplicated and collected on early-generation progeny. While the statistical methods are well established in animal and tree breeding programmes, their implementation in an operational self-pollinating crop breeding programme, in combination with OCS and index-based selection, is unique and provides new empirical insights into this strategy for breeding crops for complex, low-to-medium heritability traits.

We achieved high rates of realised genetic gain when crossing occurred among highly heterozygous early-generation progeny. This allowed short selection cycles and avoided the cost and time required to generate near-homozygous lines by selfing before selection (Watson et al. 2018). The facilities for rapid single-seed descent are not available or are not affordable to many plant breeding programmes, including this programme on common beans in Uganda. Our strategy builds on an early-generation genomic selection strategy modelled by Gaynor et al. (2017) called the population improvement component of self-pollinating crop breeding. We extend this concept to include OCS and index selection for multiple low-to-medium heritability traits, and we allow high-value inbred genotypes to be selected on merit as parents in subsequent cycles of selection. Based on our methods (Fig. 1), near-homozygous superior genotypes are quickly available for exploitation in the product development component of crop breeding (Gaynor et al. 2017).

This breeding strategy is both practical and impactful. Multivariate genomic analysis generated accurate breeding values and revealed correlated genetic relationships among traits, both favourable and detrimental. OCS and weighted selection indices were adjusted within each cycle to achieve improvement in multiple traits, based on timely adjustments to weights in the selection index in line with new knowledge of genetic correlations and changes in breeding objectives.

Predictive ability for CKT across cycles was low, despite its favourable genetic correlations with other traits. This implies that phenotyping for CKT in the majority of genotypes remains necessary in each cycle. However, prediction was improved when phenotypic data for correlated traits within the cycle were included in multivariate genomic analysis in a leave-one-trait-out approach. The inclusion of correlated phenotypic traits such as WAC, Fe and Zn in multivariate LMMs improved the predictive ability and rank correlation of GBLUPs and HBLUPs for CKT across breeding cycles. In the future, it may be possible to reduce phenotyping for CKT while continuing to phenotype Fe, Zn, WAC and SW100. The latter traits are less expensive and less time-consuming to measure, but their inclusion in multivariate LMMs also improves predictive ability for CKT in S_0_ individuals which have not been phenotyped for CKT.

A high level of genetic diversity was retained in this population through the use of optimised crossing designs in OCS. There was a small increase in mean parental coancestry from cycle 1 to cycle 2, while the rate of genetic gain for CKT was very high at -11% per year. This slow loss in genetic diversity and rapid genetic gain after 2 cycles reflects the outcomes observed in models (Cowling et al. 2017, 2019) and in a commercial canola breeding programme based on the BRIO breeding system in self-pollinating crops (Cowling et al. 2023). The accumulation of data across breeding cycles is expected to enhance predictive ability in future cycles. By leveraging correlated trait data, applying appropriate weighted selection indices through desired gains, and adopting end-user-oriented parent selection strategies for optimal cross selection with OCS, it should be possible in future to maintain the rate of genetic gain for CKT while managing trade-offs across Fe, Zn, SW100 and grain yield. These results provide support for a change in the process of breeding self-pollinating crop plants to include rapid cycles of early-generation selection based on multivariate single-step genomic analysis, an index composed of multiple economic traits and OCS in the population improvement phase.

## Supplementary Information

Below is the link to the electronic supplementary material.

Supplementary file1 (XLSX 726 KB)

## Data Availability

The authors will make available the original data to interested parties upon request.
